# Malignant transformation of residual endometriosis following hysterectomy and bilateral salpingo-oophorectomy in a female patient from a family with hereditary non-polyposis colorectal cancer

**DOI:** 10.3892/ol.2013.1182

**Published:** 2013-02-06

**Authors:** YUXUAN CHEN, YAN ZHAI, XIAOYING JIANG, ZHENYU ZHANG

**Affiliations:** 1 Beijing Chaoyang Hospital, Beijing, P.R. China; 2 Beijing Youan Hospital, Affiliated Hospital of Capital Medical University, Beijing, P.R. China

**Keywords:** malignant transformation, hereditary non-polyposis colorectal cancer, residual endometriosis

## Abstract

The aim of this study was to report a case of malignant transformation from residual endometriosis following hysterectomy and bilateral salpingo-oophorectomy in a female patient with a positive family history of ovarian and colon cancer resulting from residual endometriosis. A 42-year-old female patient from a family with hereditary non-polyposis colorectal cancer (HNPCC) diagnosed with bilateral ovarian endometriosis underwent a hysterectomy and bilateral salpingo-oophorectomy. Two years later, the patient was diagnosed with malignant ovarian cancer. Histological examination revealed an endometrioid adenocarcinoma with transitions between endometriosis and adenocarcinoma. The patient was diagnosed with ovarian endometrioid carcinoma, at FIGO stage IIC. In future, the family history of female patients with endometriosis should be collected. The association between the malignant transformation of endometriosis and HNPCC should be studied further in a research setting.

## Introduction

Endometriosis is a benign hormone-dependent condition, occurring at various degrees of the disease in 5–15% of females. The pathogenesis of endometriosis is yet to be completely understood, although studies have shown that it may cause retrograde implantation of menstrual tissue, peritoneal metaplasia and lymphatic and venous spread (1–3). These features have mixed traits of benign disease and malignancy. Patients with a long history of endometriosis have a higher risk of developing ovarian cancer (4,5). The incidence of malignant transformations ranges between 0.7 and 1.0% in patients with endometriosis (6,7). Sampson (8) first defined the following criteria for diagnosing malignant transformation of endometriosis: i) there should be a clear example of endometriosis in proximity to the tumor; ii) no other primary site of the tumor may be found; and iii) the histological appearance should be consistent with an endometrial origin (8). Scott completed Sampson’s criteria by adding the demonstration of a transition between endometriosis and malignant epithelium (9).

The present study describes a case of malignancy arising from residual endometriosis in a female patient with a family history of ovarian and colon cancer following hysterectomy and bilateral salpingo-oophorectomy. The study was approved by the Ethics Committee of Beijing Chaoyang Hospital, Beijing, China. Written informed consent was obtained from the patient.

## Case report

**Figure 1:**
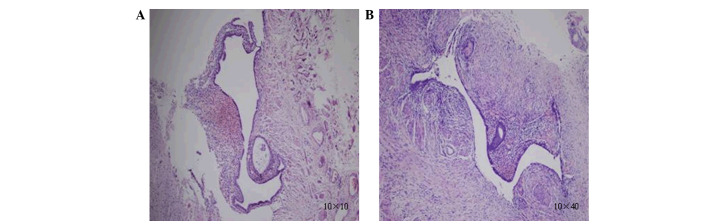
(A) Histological result from the patient’s first surgery revealed endometriosis in the right ovary and salpinx. (B) Histological results from the patient’s second surgery revealed endometriosis in the left ovary.

**Figure 2:**
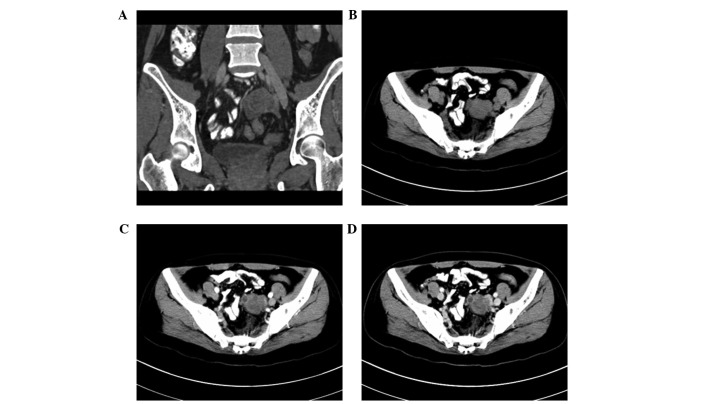
Contrast-enhanced CT image of the pelvic masses in the present case. (A) Coronal image of the pelvic masses. (B–D) A mixed density pelvic lobulated mass was observed from. Moderately non-homegeneous enhancement was observed in arterial phases. CT, computed tomography.

**Figure 3:**
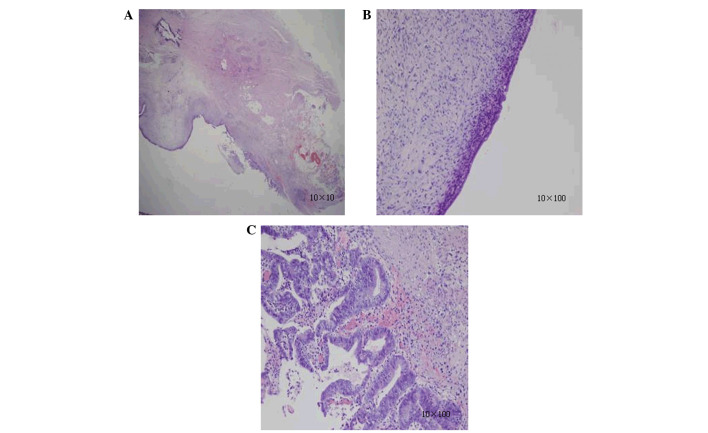
(A) Histological examination from the patient’s third surgery revealed a transition between endometriosis and endometrioid adenocarcinoma at 10×10 magnification. (B) Endometriosis in the residual left ovary at 10×100 magnification. (C) Moderately differentiated endometrioid adenocarcinoma in the residual left ovary at 10×100 magnification.

A 42-year-old female patient, para 1, was admitted on August 30, 2011, to the Department of Obstetrics and Gynecology at Beijing Chaoyang hospital (Beijing, China) due to a recent-onset pelvic cyst persisting for five months. The patient had a five-year history of ovary endometriosis and had undergone right-side laparoscopic salpingo-oophorectomy and left ovarian cystectomy for bilateral ovary endometriosis in November 2006. A histological examination showed benign endometriosis in the right ovary and salpinx (Fig. 1A) and a luteal cyst was identified in the left ovary. After the surgery, the patient received gonadotropin-releasing hormone (GnRH) agonist treatment (goserelin, 3.6 mg every 4 weeks for 6 months) and was followed up using serial cancer antigen 125 (CA125). The patient returned to the hospital three years later complaining of having hysteromyoma for 18 months with menorrhagia for 12 months. Laparoscopic surgery was performed on March 18, 2009, and an 8-cm intramural myoma and 2-cm cystic mass were identified in the left ovary. The left ovary and salpinx were markedly adhered to the sigmoid colon. Laparoscopy with hystrorectomy and left salpingo-oophorectomy were performed. The histology of the left ovarian cyst exhibited endometriosis (Fig. 1B). Hormone replacement therapy was not selected following surgery. The patient was followed up using CA125 level and pelvic ultrasonography which remained normal five months after surgery. The following hormone levels were tested after the surgery: serum estrogen, follicle stimulating hormone (FSH) and luteinzing hormone (LH; estrogen 2 127.14 pg/ml, FSH 7.44 IU/l, LH 5.86 IU/l). The serum CA125 level was elevated to 119.8 U/ml (normal <35 U/ml). A contrast-enhanced computed tomography (CT) scan was then performed to aid in identifying the reasons for the abnormal test results. The scan showed a mixed-density pelvic lobulated mass measuring 4.2×3.3×3.8 cm on the left side with an irregular surface and exhibiting moderately nonhomegeneous arterial enhancement (Fig. 2). The patient then underwent laparoscopy, which identified a 6×7-cm mass adhering to the left pelvic wall and colon. Subsequently, the adhesion was released and the mass was resected for a frozen biopsy, which revealed adenocarcinoma of the residual left ovary. Laparoscopic staging surgery was then performed, including excision of the tumor mass, omentectomy, appendectomy and pelvic/paraaortic lymphadenectomy. Histological examination following surgery showed that part of the residual normal ovary tissue had moderately differentiated into endometrioid adenocarcinoma directly arising from the residual endometriosis site, without pelvic and paraaortic lymph node involvement. A transition region between endometriosis and endometrioid adenocarcinoma was also observed (Fig. 3). The patient was diagnosed with ovarian endometrioid adenocarcinoma at FIGO stage IIC. The postoperative chemotherapy consisted of 165 mg/m^2^ paclitaxel and AUC 5 of carboplatin.

**Figure 4:**
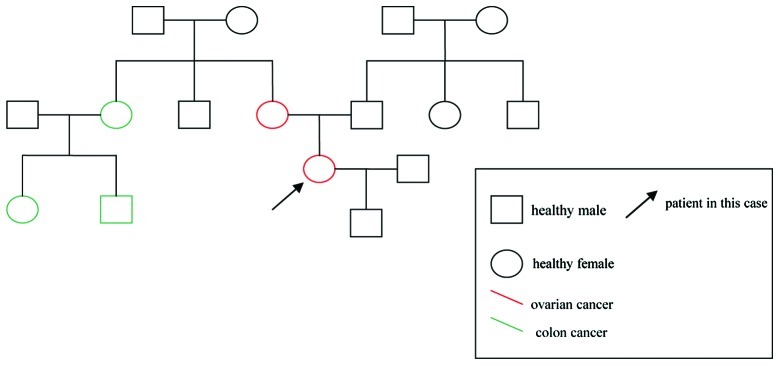
Pedigree of the patient’s family with a poor tumor history. Family members in red were diagnosed with ovarian cancer. Family members in green were diagnosed with colon cancer. Four family members were diagnosed before the age of 50 (the patient at 42 years old, the patient’s mother at 51 years old, the patient’s mother’s sister at 45 years old and the daughter and son of the sister at the ages of 38 and 40, respectively).

The patient’s family history for three generations is presented in Fig. 4. Three cases of colon cancer and one case of ovarian cancer were identified on the maternal side of the family. No cases were identified on the paternal side.

## Discussion

It has been reported that endometrial lesions in the ovary have the potential for malignancy (10–12). In the present case, endometrioid adenocarcinoma was shown to arise from endometriosis and the transition between endometriosis and endometrioid carcinoma was confirmed and met the criteria of Sampson and Scott (8,9). This case was a female patient with a family history of ovarian and colon cancer who underwent a malignant transformation two years after pelvic clearance surgery, hysterectomy and bilateral salpingo-oophorectomy.

In the present case, five family members were diagnosed with cancer, two with ovarian cancer and three with colon cancer, as shown in Fig. 4. The results of these cases fulfill the Amsterdam II criteria and the Bethesda guidelines (Diagnosis criteria for HNPCC) (13). A diagnosis of HNPCC should be considered (Table I). Women with HNPCC have an increased risk of gynaecological cancer (14). Among women with HNPCC, 20–60% may develop endometrial cancer compared with 3% of the general population. Ovarian cancer occurs in 10–20% women with HNPCC (15). Additionally, according to Matalliotakis *et al*, there is a relative risk for women with endometriosis and a positive family history of ovarian and colon cancer including first- and second-degree relatives (16). The study indicated that HNPCC may be associated with gynaecological cancer. However, little evidence has been reported on the association between the malignant transformation of endometriosis and HNPCC. HNPCC is an autosomal dominantly inherited cancer disorder (Fig. 4) and has been demonstrated to be caused by the inherited mutation of genes such as hMSH2, hMLH1, PMS1, PMS2 and hMSH6 (17,18). The HNPCC gene mutations continue to develop and accumulate within neoplastic but not normal tissue (19). However, associated studies have suggested that the malignant transformation of endometriosis may be induced by loss of heterozygosity (LOH) events on certain chromosomes such as the PTEN gene situated on chromosome 10q23.3 (20,21). Whether certain special HNPCC gene mutations are involved in the malignant transformation of endometriosis remains unknown. In the present case, the malignant transformation of endometriosis may have arisen from the incomplete excision of the left ovary in the patient’s second surgery. The left ovary was identified as being markedly adhered to the colon during the patient’s second surgery. A meticulous excision was difficult to perform (22,23), which may have resulted in a trace amount of residual left ovary. The blood supply of the residual ovary tissue may account for the formation of collateral circulation (24). According to the levels of female hormones, the residual ovary was able to maintain normal endocrine function (23,24). It has been reported that hyperestrogenism is closely associated with the malignant transformation of endometriosis (4,25). In previously reported cases, women who underwent pelvic clearance surgery and later underwent estrogen replacement therapy had a markedly higher risk of malignant extra-gonadal transformation (4). Hormonal factors may be crucial in the origin of endometriosis and the development of malignant transformation (4,20,26). As discussed previously, the incomplete excision of the left ovary may have resulted in normal levels of serum estrogen. Whether the normal levels of female hormones contribute to the malignant transformation of ovary endometriosis has yet to be proved.

As mentioned previously, we suggest that an accurate family history should also be obtained from women with endometriosis. As for women with HNPCC, hysterectomy and bilateral salpingo-oophorectomy should be considered as the patient’s first surgical treatment. Further studies of malignant endometriosis-associated gene detection in HNPCC should also be performed.

## Figures and Tables

**Table I:** Clinical criteria for HNPCC.

Name	Criteria
Amsterdam	At least three relatives with CRC; all the following criteria should be present: One should be the first-degree relative of the other two At least two successive generations should be affected At least one CRC should be diagnosed before the age of 50 Familial adenomatous polyposis should be excluded
Amsterdam II	At least three relatives with an HNPCC-associated cancer (CRC, cancer of the endometrium, small bowel, ureter or renal pelvis); all the following criteria should be present: One should be the first-degree relative of the other two At least two successive generations should be affected At least one CRC should be diagnosed before the age of 50 Familial adenomatous polyposis should be excluded
Bethesda (modified)	Individuals with cancer in families that fulfil the Amsterdam criteria Individuals with two HNPCC-related cancers, including synchronous or metachronous CRCs or associated extra-colonic cancers Individuals with CRC and a first-degree relative with CRC and/or HNPCC-related extracolonic cancer and/or colorectal adenoma; one of the cancers diagnosed at <50 years and the adenoma diagnosed at <40 years Individuals with CRC or endometrial cancer diagnosed at <50 years Individuals with right-sided CRC with an undifferentiated pattern (solid/cribriform) on histopathology diagnosed at <50 years Individuals with signet-ring-cell-type CRC diagnosed at <50 years Individuals with adenomas diagnosed at <40 years

HNPCC, hereditary non-polyposis colorectal cancer; CRC, colorectal cancer.

## References

[1] Olive DL, Schwartz LB (1993). Endometriosis. N Engl J Med.

[2] Hacker NF, Gambone JF, Hobel CJ (2010). Hacker and Moore’s Essentials of Obstetrics and Gynecology.

[3] Czernobilsky B, Fox H, Wells M (1995). Endometriosis. Haines and Taylor Obstetrical and Gynaecological Pathology.

[4] Benoit L, Arnould L, Cheynel N, Diane B, Causeret S, Machado A, Collin F, Fraisse J, Cuisenier J (2006). Malignant extraovarian endometriosis: a review. Eur J Surg Oncol.

[5] Munksgaard PS, Blaakaer J (2011). The association between endometriosis and gynecological cancers and breast cancer: a review of epidemiological data. Gynecol Oncol.

[6] Nishida M, Watanabe K, Sato N, Ichikawa Y (2000). Malignant transformation of ovarian endometriosis. Gynecol Obstet Invest.

[7] Corner GW, Hu CY, Hertig AT (1950). Ovarian carcinoma arising in endometriosis. Am J Obstet Gynecol.

[8] Sampson JA (1925). Endometrial carcinoma of the ovary, arising in endometrial tissue in that organ. Arch Surg.

[9] Scott RB (1953). Malignant changes in endometriosis. Obstet Gynecol.

[10] Brinton LA, Gridley G, Persson I, Baron J, Bergqvist A (1997). Cancer risk after a hospital discharge diagnosis of endometriosis. Am J Obstet Gynecol.

[11] Aris A (2010). Endometriosis-associated ovarian cancer: a ten-year cohort study of women living in the Estrie Region of Quebec, Canada. J Ovarian Res.

[12] Xu B, Hamada S, Kusuki L, Itoh R, Kitawaki J (2011). Possible involvement of loss of heterozygosity in malignant transformation of ovarian endometriosis. Gynecol Oncol.

[13] Vasen HF, Mecklin JP, Khan PM, Lynch HT (1991). The International Collaborative Group on Hereditary Non-Polyposis Colorectal Cancer (ICG-HNPCC). Dis Colon Rectum.

[14] Sharma A, James M, Donaldson A, Fox R (2001). Hereditary non-polyposis colorectal cancer syndrome: combined risk of gastrointestinal and gynaecological cancer. BJOG.

[15] Allen BA, Terdiman JP (2003). Hereditary polyposis syndromes and hereditary non-polyposis colorectal cancer. Best Pract Res Clin Gastroenterol.

[16] Matalliotakis IM, Cakmak H, Krasonikolakis GD, Dermitzaki D, Fragouli Y, Vlastos G, Arici A (2010). Endometriosis related to family history of malignancies in the Yale series. Surg Oncol.

[17] Peltomäki P (2012). Mutations and epimutations in the origin of cancer. Exp Cell Res.

[18] Vasen HF, Watson P, Mecklin JP, Lynch HT (1999). New clinical criteria for hereditary nonpolyposis colorectal cancer (HNPCC, Lynch syndrome) proposed by the International Collaborative Group on HNPCC. Gastroenterology.

[19] Jass JR, Stewart SM, Stewart J, Lane MR (1994). Hereditary non-polyposis colorectal cancer - morphologies, genes and mutations. Mutat Res.

[20] Sato N, Tsunoda H, Nishida M, Morishita Y, Takimoto Y, Kubo T, Noguchi M (2000). Loss of heterozygosity on 10q23.3 and mutation of the tumor suppressor gene PTEN in benign endometrial cyst of the ovary: possible sequence progression from benign endometrial cyst to endometrioid carcinoma and clear cell carcinoma of the ovary. Cancer Res.

[21] Xu B, Hamada S, Kusuki I, Itoh R, Kitawaki J (2011). Possible involvement of loss of heterozygosity in malignant transformation of ovarian endometriosis. Gynecol Oncol.

[22] Minelli L, Ceccaroni M, Ruffo G, Bruni F, Pomini P, Pontrelli G, Rolla M, Scioscia M (2010). Laparoscopic conservative surgery for stage IV symptomatic endometriosis: short-term surgical complications. Fertil Steril.

[23] Fedele L, Bianchi S, Zanconato G, Bergamini V, Berlanda N, Carmignani L (2005). Long-term follow-up after conservative surgery for bladder endometriosis. Fertil Steril.

[24] Achard JM, Fournier A, Mazouz H, Caride VJ, Penar PL, Fernandez LA (2001). Protection against ischemia: a physiological function of the renin-angiotensin system. Biochem Pharmacol.

[25] Nezhat F, Datta MS, Hanson V, Pejovic T, Nezhat C, Nezhat C (2008). The relationship of endometriosis and ovarian malignancy: a review. Fertil Steril.

[26] Somigliana E, Vigano P, Parazzini F, Stoppelli S, Giambattista E, Vercellini P (2006). Association between endometriosis and cancer: a comprehensive review and a critical analysis of clinical and epidemiological evidence. Gynecol Oncol.

